# A Green Film-Forming Investigation of the Edible Film Based on Funoran: Preparation, Characterization, and the Investigation of the Plasticizer Effects

**DOI:** 10.3390/foods11192971

**Published:** 2022-09-23

**Authors:** Zhisheng Zhang, Han Wang, Sohail Khan, Ying Shu, Tieqiang Liang

**Affiliations:** College of Food Science and Technology, Hebei Agricultural University, Lekai South Avenue, Baoding 071000, China

**Keywords:** funoran, edible films, film-forming property, plasticizer effects

## Abstract

In this study, an edible film based on funoran was developed. Moreover, the effects of plasticizers (glycerol, xylitol, and sorbitol) on the physicochemical properties of the funoran films were also investigated. The interactions between plasticizers and funoran molecules of the film-forming system were characterized by Fourier transform infrared spectroscopy, thermogravimetric analysis, and scanning electron microscopy. The results showed that the addition of plasticizers altered and broke the initial complex entangled structures of funoran molecular chains. Funoran films containing plasticizers were compatible, homogeneous, and dense, exhibiting good thermal stability below 100 °C. With the addition of plasticizers, the elongation at break, oxygen permeability, light transmittance, and water vapor permeability increased, but the tensile strength decreased. It was found that a glycerol addition of 40% was most suitable for commercial applications. All the results revealed the excellent film-forming properties of funoran, indicating that the prepared funoran films have tremendous potential for packaging applications.

## 1. Introduction

Food packaging materials are usually used to isolate foods from their surroundings, control moisture and gas exchange, and reduce oxidation reactions [1], thus avoiding the loss of desired compounds (flavor volatiles) and extending the shelf life [2]. In food packaging applications, petroleum-based polymers and plastics have become increasingly popular due to their low cost, good mechanical properties, and high practicality. Moreover, the application of plastics in food packaging has exceeded half of the total materials, ranking first among various packaging materials. However, these traditional polymers and plastics are not biodegradable and not renewable, making plastic pollution a global problem now.

Compared to synthetic plastic packaging, biodegradable packaging is safe, nontoxic, biodegradable, renewable, and bioavailable [3,4], causing no pollution to the environment [5,6]. However, the poor mechanical properties and high production costs of biodegradable films limit their commercial application [7]. Therefore, the development of new biological materials has become a current research hotspot in the field of biodegradable film materials.

Among various biodegradable materials, polysaccharides are nontoxic and abundant. Their tight network structure provides excellent barrier properties to CO_2_ and O_2_ without adding plasticizers [8]. Biodegradable films made from polysaccharides such as cellulose, starch, chitosan, alginate, and pectin have been developed to replace traditional plastic packaging [9,10,11]. However, most polysaccharides exhibit shrinkage when water evaporates or dries quickly, resulting in film defects such as brittleness, cracking, or curling. In order to solve these defects, green plasticizers are usually added to increase the flexibility and ductility of the film by breaking the hydrogen bonds between polymer molecules and reducing intermolecular forces [12,13]. In addition, many plasticizers, such as glycerol (G), xylitol (X), and sorbitol (S), have been used to increase the toughness of bio-based films [14,15,16].

**Figure 1:**
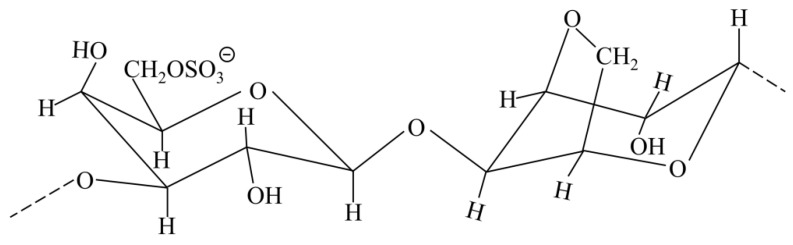
The main polar structural formula of funoran.

As one of the polysaccharides, funoran is less frequently used as a film-forming substrate for preparing biodegradable films. It is a heterogeneous galactose sulfated polysaccharide with 3,6-anhydro-α-L-galactose and β-D-galactose-6-sulfate extracted from the date palm red algae repeating units, and its structure is shown in Figure 1 [17,18,19]. Through the investigation, most of the research on furan is currently the extraction method and structural characteristics [17,18,19]. However, Ju and Song [17] prepared an antioxidant film by mixing funoran extracted from red algae with onion peel extraction and found that it exhibited high radical scavenging activities. They were flexible and could be used as active biodegradable packaging, but the film-forming properties of funoran were not investigated.

In this study, the film-forming properties of funoran were investigated. Plasticizers including G, X, and S were used to prepare edible funoran films. Fourier transform infrared (FTIR) spectroscopy, scanning electron microscopy (SEM), thermogravimetric analysis (TGA), moisture content, moisture absorption, oxygen permeability (OP), water vapor permeability (WVP), mechanical properties, and opacity were used to characterize the funoran film. This study could lead to the development of a new scientific edible substrate for the production of edible films.

## 2. Materials and Methods

### 2.1. Experimental Materials and Reagents

Funoran (Food grade, FG) was purchased from Shandong Ruisite Biology Science and Technology Co., Ltd. (Shandong, China). Glycerol (G) (Analytical reagent, AR), Xylitol (X) (Biological reagent, BR), and sorbitol (S, BR) were purchased from Tianjin Kermel Chemical Reagent Co., Ltd. (Tianjin, China). Anhydrous calcium chloride (AR) was purchased from Tianjin Tianda Chemical Experimental Factory (Tianjin, China). Anhydrous potassium carbonate (AR) was purchased from Fuchen (Tianjin) Chemical Reagent Co., Ltd. (Tianjin, China) to prepare a constant humidity solution with a relative humidity of 43%. Sodium chloride (GR) was purchased from Sinopharm Chemical Reagent Co., Ltd. (Shanghai, China) to prepare a constant humidity solution with a relative humidity of 75%.

### 2.2. Preparation of Funoran Films

Based on the results of the preliminary experiments, the film-forming solution prepared by funoran and distilled water in a ratio of 6:700 (*w*/*v*) was easier to stir and less prone to air bubbles. In addition, funoran film without plasticizer was fragile and difficult to peel into a complete film. Therefore, G, X, and S were used as plasticizers in funoran films. Because films containing 20% plasticizer still showed fragility and difficulty in peeling, plasticizers of 20–50% (*w*/*w*, funoran basis) were employed.

The preparation of funoran film was performed using the casting method as follows [20]:

Firstly, 6 g of funoran powder was added to 700 mL of distilled water and stirred for 30 min at 60 °C with a speed of 900 rpm. Afterward, 1.2 g, 1.8 g, 2.4 g, and 3.0 g of plasticizers were transferred to the above solution. After stirring for 15 min, the prepared film-forming solution was moved to a vacuum drying oven to defoam for 2 h. The film-forming solution was then poured into an acrylic plate (26 cm × 26 cm × 4 cm) and dried in an oven at 60 °C for 24 h. Finally, the dried funoran film was peeled and stored at a constant humidity of 43% for 12 h prior to performance testing. The prepared funoran film was labeled as G0–G50, X0–X50, and S0–S50. G, X, and S are abbreviations for glycerol, Xylitol, and sorbitol, and 0–50 in the labels indicate the addition amount of plasticizer is 0–50% (*w*/*w*, funoran basis).

### 2.3. Structural Characterization of Funoran Films

#### 2.3.1. FTIR Test

FTIR (ATR model) spectra of funoran films were recorded using the FTIR spectrophotometer (Nicolet iS5, Thermo Fisher Scientific, Waltham, MA, USA). Based on the obtained spectra, the chemical structural changes in the funoran films were analyzed. The tests were performed with a wavelength range of 4000–600 cm^−1^ (the resolution was 4 cm^−1^).

#### 2.3.2. TGA Test

The thermal stability of funoran films was evaluated by TGA using a TG 209 F3 Tarsus series thermal weight loss analyzer (NETZSCH). This process was protected by high-precision N_2_ (20 mL/min) with a temperature range of 25–600 °C (10 °C/min) [21,22].

#### 2.3.3. SEM Test

The cross-sectional morphology of funoran films was observed on a floor-standing scanning electron microscope (Prisma E, Philips-FEI Co., Eindhoven, The Netherlands). Before testing, the funoran films underwent brittle fracture in liquid nitrogen, followed by the fixation on a sample holder with conductive adhesive. After coating the samples with gold, 5 kV accelerating voltage was applied, and 2000× was selected as the magnification for observation [23].

### 2.4. Physical Properties Test

#### 2.4.1. Film Thickness Test

The film thickness was measured with a digital micrometer (SanLiang Digimatic Micrometer, Shanghai, China) and recorded as the average of values measured at five random points.

#### 2.4.2. Mechanical Properties Test

Tensile strength (TS) and elongation at break (EB) are the main indicators of the mechanical properties of the film. The mechanical properties of funoran films were measured using a food texturizer (TMS-Pilot, OHAUS (Shanghai) Instruments Co.) referring to the Chinese GB/T 1040.3-2006 standard. The funoran films were cut into 15 mm × 70 mm strips and tested under a strain rate of 200 mm/min at 25 °C. TS and EB were calculated according to Equations (1) and (2), respectively. The results were reported on an average of six tests. Then, the TS and EB values were calculated according to the following equations:

TS = F/(W × H)(1)

EB = [(l − l_0_)/l_0_] × 100 (2)

where TS and EB are the tensile strength (MPa) and elongation at break (%), respectively; F is the maximum load applied to the strips (N); W is the width of the strips (mm); H is the average thickness of the strips (mm); l and l_0_ are the length of the strips at break and initially (mm), respectively.

#### 2.4.3. OP Test

The OP test of the funoran films referred to the Chinese GB/T 1038-2000 standard. Prior to testing, a 97 mm diameter circular sample was taken with a sampler and then tested for oxygen transmission (Q_g_) by the differential pressure method on a gas permeation test system (C130H, Labthink Instruments Co., Ltd., Boston, MA, USA) at 25 °C. The flow rate of both N_2_ and O_2_ was 10 mL/min, the test humidity was 0%, and the test area was 0.005 m^2^. The results were recorded on an average of six tests, as calculated through the following equation:

OP = 1.1574 × 10^−9^Q_g_ × H (3)

where OP is the gas transmission rate (cm^3^∙cm/(m^2^∙d∙0.1 MPa)); H is the average thickness of the sample (cm).

#### 2.4.4. WVP Test

The WVP test for funoran films was referenced to the Chinese GB 1037-88 standard using the method described by Farhan and Hani [24]. Firstly, a moisture-permeable cup containing completely dry CaCl_2_ pellets (0% humidity) was sealed with funoran films by hot-melt adhesive. The cup was stored in a sealed apparatus containing a saturated NaCl solution at 25 °C and 75% humidity, and the osmotic pressure of the container was 1753.55 Pa. Afterward, the permeable cups were weighed at regular intervals for 6 h. The WVP was calculated according to Equation (4):

WVP = (Δm × H)/(Δt × S × P)(4)

where WVP is the water vapor permeability (g/(s∙m∙Pa)); ∆m is the water vapor transmission (g); H is the average thickness of the film (mm); S is the test area (0.00196 m^2^); ∆t is the test time (s); P is the osmotic pressure of a saturated solution of NaCl (1753.55 Pa).

#### 2.4.5. Light Transmittance Test

The light transmittance of funoran films was performed on a UV-Visible spectrophotometer (UV-2600, Shimadzu) according to the method described by Shivangi et al. [25]. Funoran films were cut into 50 mm × 50 mm pieces and tested in a wide wavelength range (200–800 nm).

### 2.5. Statistical Analysis

All experimental data were processed by ANOVA in SPSS 23 software, and Duncan’s test (*p* < 0.05) was conducted to compare the means of the results. Results for each test (samples in triplicate or more) were reported as mean ± root mean squared error. Small letters in the figures indicate significant differences between funoran films at the (*p* < 0.05) level. All graphs were plotted using Origin software (2019b).

## 3. Results and Discussion

### 3.1. FTIR Analysis

**Figure 2:**
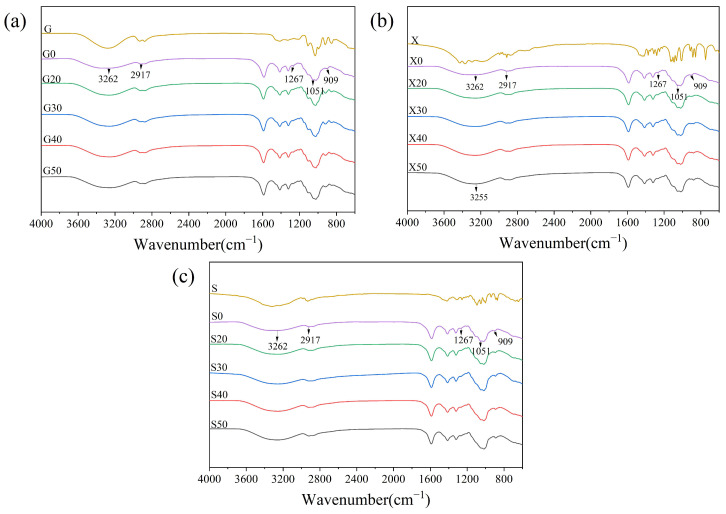
Spectra of the funoran films with different additions of G (**a**), X (**b**), and S (**c**).

The interactions in the funoran films can be reflected by the shifts and intensity variations of the characteristic peaks of FTIR spectra. Due to strong interactions between different polymers, the bands in compatible blended systems deviate significantly from each polymer component [23,26]. As shown in Figure 2, the broadband of the unplasticized funoran film (G0, X0, and S0) at 3262 cm^−1^ is free, intramolecular, and intermolecular hydrogen-bonded O-H stretching vibrations [27]. The band at 2917 cm^−1^ is attributed to the C-H antisymmetric stretching vibration in the methylene [28]. The bands at 1267 cm^−1^, 1051 cm^−1^, and 909 cm^−1^ are attributed to the O=S=O stretching vibration of the sulfate ester group, the C-O-C stretching vibration in 3, 6-endo-D-galactose, and the C-O-S stretching vibration in 6-sulfate-β-D-galactose, respectively [17,18]. In particular, the bands at 1267 cm^−1^, 1051 cm^−1^, and 909 cm^−1^ are the characteristic peaks of funoran [17].

The FTIR spectra of the G and G-plasticized funoran films are shown in Figure 2a. The band intensity at 3262 cm^−1^ and 2917 cm^−1^ increased with G, and the bands were slightly red-shifted. When the addition of plasticizer exceeded 30%, the bands at these two positions showed a greater blue shift, indicating a change of hydrogen bonds in the funoran film. The addition of G altered and rebuilt the three types of hydrogen bonds within the film-forming system (intermolecular hydrogen bonds between funoran molecules, hydrogen bonds formed between funoran molecules and G molecules, and intermolecular hydrogen bonds between G molecules). Due to the formation of new hydrogen bonds between G and funoran molecules, the blue shift of the O-H band was small at low G addition, reducing the free -OH in the film-forming system. With the increase of G addition, the blue shift of the O-H band increased as the addition of G broke the hydrogen bonds among funoran molecules [15]. The results indicated that new hydrogen bonding interactions were formed between funoran and G molecules with good compatibility.

The FTIR spectra of X and X-plasticized funoran films are shown in Figure 2b. It can be seen that the addition of X red-shifted the band of funoran film from 3262 cm^−1^ to 3255 cm^−1^, indicating that the hydrogen bonds among funoran molecules were disrupted by X. When the X addition exceeded 30%, the band slightly blue-shifted. At this point, the overall shift is larger than that of G-plasticized funoran films, indicating that the addition of X broke the hydrogen bonds between funoran molecules and formed new bonds between funoran and X molecules [29,30]. The results are similar to the addition of G.

The FTIR spectra of S and S-plasticized funoran films are shown in Figure 2c. It can be seen that the structural changes of the S-plasticized funoran films were similar to those of the G and X-plasticized funoran films, with good compatibility between funoran and S molecules.

### 3.2. TGA Analysis

**Figure 3:**
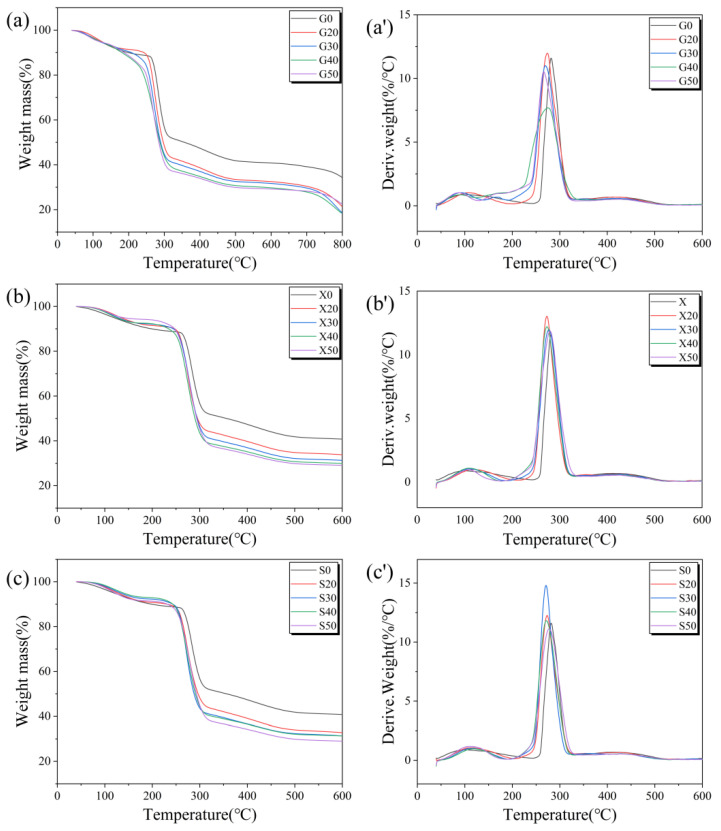
TG and DTG curves of the funoran films with different additions of G (**a**,**a’**), S (**b**,**b’**), and X (**c**,**c’**).

As can be seen in Figure 3 for the funoran films without plasticizers (G0, S0, and X0), there are two significant mass loss peaks for these films. The first mass loss peak is the loss of water at around 103.62 °C, and the second is around 281.86 °C, which is the main decomposition peak of funoran. This result is consistent with the thermal degradation stage corresponding to the vegetable gum-based films mentioned by Razavi et al. [31] and Martins et al. [32].

The TG and DTG curves for the G-plasticized funoran films are shown in Figure 3a,a’, in which three peaks of mass loss can be observed. The first peak is the mass loss peak of adsorbed water near 103.62 °C [33]. The peak is narrower, and the mass-loss rate increases compared to the funoran film without plasticizer. The reason might be that the addition of plasticizer increased the distance between funoran molecular chains, resulting in increased water mobility [21]. The second peak at 165.40 °C is the pyrolysis peak of G [27], which increases with the G addition. The third peak is the decomposition peak of funoran near 281.86 °C. The highest degradation rate was observed at this stage, indicating a rapid decrease in the mass of the funoran film. This peak shifted slightly toward the lower temperature compared to the funoran film without plasticizer, but the mass loss rate decreased. The above results indicate that G destabilized the interactions between funoran molecular chains, but the thermal stability of the G-plasticized films remains excellent due to the decomposition temperature above 100 °C.

The TG and DTG curves for the X-plasticized funoran films are shown in Figure 3b,b’, in which two mass loss peaks can be observed. The first is the mass loss peak of adsorbed water near 121.32 °C. Due to the hydrophilicity of xylitol, it shifted slightly toward the high temperature compared with the funoran film without plasticizer. The second is the decomposition peak of X and funoran near 273.39 °C, demonstrating the good compatibility of X and funoran. In addition, the second peak slightly shifted toward a lower temperature compared with the funoran film without plasticizer, but the mass loss rate of the film increased. The above results suggest that the addition of X slightly destroyed the thermal stability of the funoran films.

The TG and DTG curves for the S-plasticized funoran films are shown in Figure 3c,c’. It can be observed that the curves of S-plasticized funoran films are similar to that of X-plasticized funoran films. The first peak near 106.54 °C is the mass loss peak of adsorbed water, which is essentially no transfer compared to the funoran film without plasticizer. The second peak near 273.00 °C is the decomposition peak of S and funoran, which shifted slightly towards the lower temperature compared to the funoran film without plasticizer.

The above comparison reveals that the thermal stability of the funoran film was slightly reduced by the addition of plasticizer, implying that the interactions between plasticizer and funoran molecules destroy the tight molecular structure among funoran molecules. As a result, the thermal stability of the funoran films was reduced [33]. These results show that the addition of plasticizers altered the initial structure of the funoran molecular chains. Furthermore, all the funoran films remain stable at temperatures below 100 °C, indicating that all of them can be used as food packaging.

### 3.3. Cross-Sectional Surface Observation

**Figure 4:**
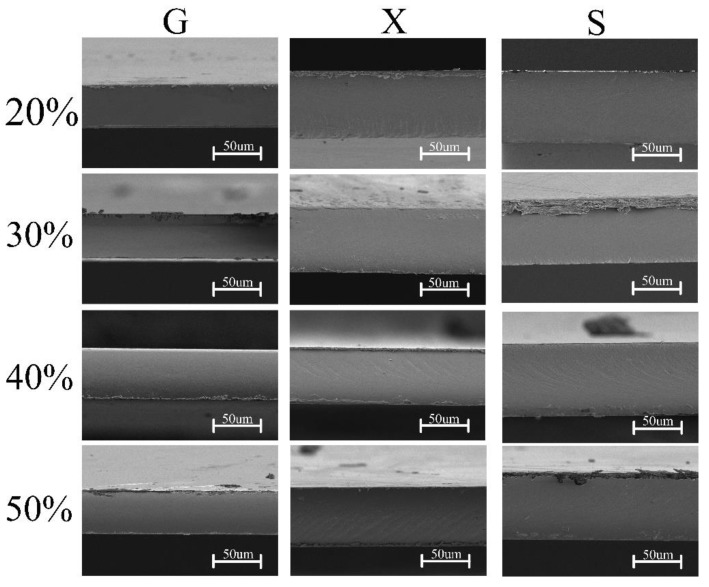
Cross-sectional surface morphologies of the funoran film with different additions of plasticizers.

The cross-sectional surface morphology of the funoran films is displayed in Figure 4. The smooth and featureless cross-sectional surfaces suggest that the plasticizers are compatible with funoran, which is consistent with the FTIR results.

**Figure 5:**
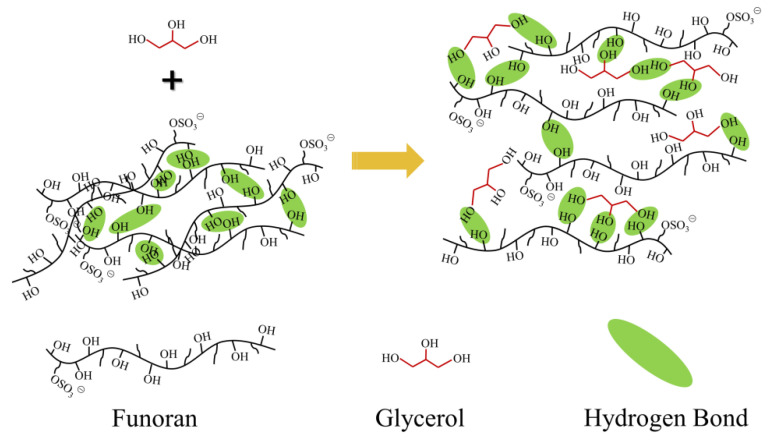
Film-forming mechanism diagram of the funoran films.

The cross-sectional surface morphologies of the S-plasticized funoran films show tiny pores due to air bubbles in the film-forming system. This phenomenon has been reported in previous studies [34,35]. The cross-sectional surface of the G-plasticized funoran films is smooth and homogeneous, while many line textures occurred on the surface of X and S-plasticized funoran films. The line texture was due to the stress effects during the brittle fracture of the funoran films in liquid nitrogen, consistent with the mechanical property results. In general, the dense structures of funoran cross-sectional surface indicate good compatibility of plasticizers with funoran, demonstrating that funoran has good film-forming properties. Based on the above analysis, the film-forming mechanism can be observed in Figure 5 when selecting G-plasticized funoran film as an example.

### 3.4. Mechanical Properties Analysis

**Figure 6:**
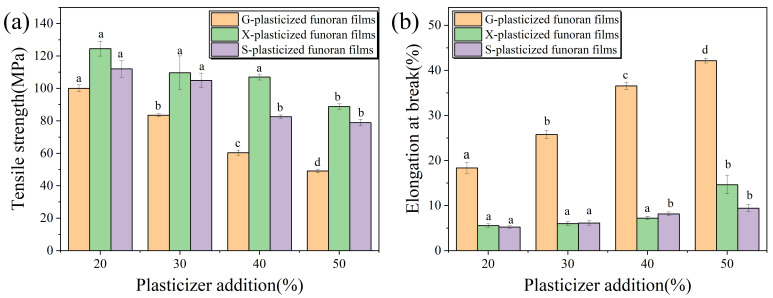
Effects of G, X and S addition on the TS (**a**) and EB (**b**) of the funoran films. The different superscript letter (a–d) indicates significant difference

The unplasticized funoran film could not be peeled due to its high brittleness. Therefore, the TS and EB values were only measured for the plasticized funoran films, as shown in Figure 6 and Table 1.

The effects of G on the mechanical properties of the funoran films are shown in Figure 6 and Table 1. As the G addition increased from 20% to 50%, the TS value decreased from 99.98 MPa to 49.10 MPa, while the EB value increased from 18.35% to 42.14%. These results show that increasing the G addition could improve the flexibility of the funoran films. The reason could be that the elongation of the G-plasticized funoran film was influenced by the mobility of molecular chains [35], and the addition of G broke the intermolecular hydrogen bonds and the complex entanglement structures between funoran molecular chains. The increased amount of -OH also increased the number of hydrogen bonds in the funoran film-forming system [33]. Therefore, the addition of G decreased the intermolecular forces and increased the mobility of funoran molecular chains [36]. In addition, the presence of G increased the water content in the film-forming system, affecting the mechanical properties of the funoran films [37]. The large number of water molecules absorbed by G in the funoran films can also function as a plasticizer, reducing the TS value and increasing the EB value [38]. Similar results can be found in the studies of Lim et al. [39] and Razavi et al. [31].

It can be seen in Figure 6 and Table 1 that as the X addition increased from 20% to 50%, the TS value decreased from 124.48 MPa to 88.77 MPa, while the EB value increased from 5.57% to 14.62%. Compared to G-plasticized funoran films, the X-plasticized funoran films have great brittleness and poor toughness during measurement.

Figure 6 and Table 1 shows that as the S addition increased from 20% to 50%, the TS value decreased from 112.04 MPa to 78.90 MPa, while the EB value increased from 5.25% to 9.40%. The overall TS values are higher for the S-plasticized funoran films than for the G-plasticized funoran films, consistent with the results of Hazrati et al. [33]. However, the S-plasticized funoran films had the lowest EB among the three plasticized funoran films and exhibited poor flexibility and mechanical properties.

In summary, the EB values of the funoran films show an increasing trend with the increasing plasticizer addition, while the TS values follow the opposite trend. These results are due to the disrupted interactions between funoran molecular chains by adding plasticizers, which increased the mobility of the funoran molecular chains [23]. The X- and S-plasticized funoran films exhibit higher TS values than G-plasticized funoran films, but their EB values are lower. The reason is that the smaller molecules of G can be more easily interspersed into the complex structures formed by funoran molecular chains, making the funoran films a denser structure. Deng et al. [40] also demonstrated that low-molecular plasticizers had better plasticization effects. The comprehensive comparison demonstrated that the mechanical properties of G-plasticized funoran films are superior, and the 40% G addition is most suitable for commercial applications.

### 3.5. OP Analysis

**Figure 7:**
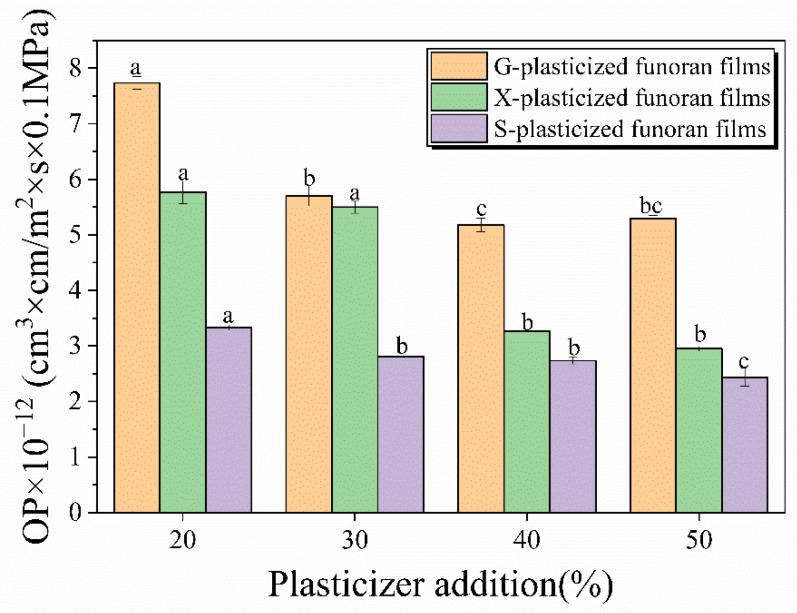
Effects of G, X and S addition on oxygen barrier property of the funoran films. The different superscript letter (a–c) indicates significant difference.

The effects of G, X, and S addition on oxygen barrier properties of the funoran films are shown in Figure 7 and Table 1. The OP values of the funoran films show a decreasing trend as the plasticizer addition increased from 20% to 50%. The OP values of the G-, X- and S-plasticized funoran films decreased from 7.73 × 10^−12^ (cm^3^∙cm)/(m^2^∙s∙0.1 MPa), 5.76 × 10^−12^ (cm^3^∙cm)/(m^2^∙s∙0.1 MPa), and 3.33 × 10^−12^ (cm^3^∙cm)/(m^2^∙s∙0.1 MPa) to 5.29 × 10^−12^ (cm^3^∙cm)/(m^2^∙s∙0.1 MPa), 2.95 × 10^−12^ (cm^3^∙cm)/(m^2^∙s∙0.1 MPa), and 2.44 × 10^−12^ (cm^3^∙cm)/(m^2^∙s∙0.1 MPa), respectively.

The comparison showed that the OP values of G-plasticized funoran films were higher than that of the X- and S-plasticized funoran films. The small molecular size of G is more easily interspersed into the complex entangled structures between funoran molecular chains, which improves the chain mobility of the funoran molecular chains and thus decreases the OP values [15]. Overall, the oxygen permeability of the funoran films with three plasticizers is good. With the increase of plasticizer, the -OH amounts in the film-forming system increased, preventing oxygen penetration through new hydrogen bonds that reform a tight structure between funoran and plasticizer.

### 3.6. WVP Analysis

WVP is a significant factor in the evaluation of food packaging. The food packaging with lower WVP can reduce the moisture exchange between the external environment and food, thus inhibiting the reproduction and growth of microorganisms and extending the shelf life of food [21].

**Figure 8:**
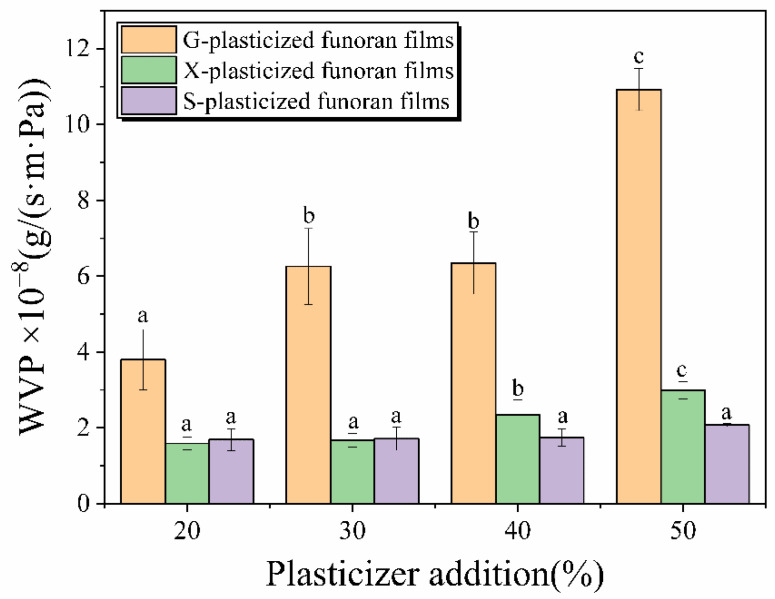
Effects of G, X and S addition on water vapor barrier property of the funoran films. The different superscript letter (a–c) indicates significant difference.

Figure 8 and Table 1 shows that the WVP values of G-, X-, and S-plasticized funoran films increased as the plasticizer addition increased from 20% to 50%. The WVP values increased from 3.80 × 10^−8^ g/(s∙m∙Pa), 1.59 × 10^−8^ g/(s∙m∙Pa), and 1.69 × 10^−8^ g/(s∙m∙Pa) to 1.09 × 10^−7^ g/(s∙m∙Pa), 2.99 × 10^−8^ g/(s∙m∙Pa), and 2.08 × 10^−8^ g/(s∙m∙Pa), respectively.

Among different films, the G-plasticized funoran films have consistently higher WVP values than X- and S-plasticized funoran films, while the difference between the X- and S-plasticized funoran films is slight. It may be due to the greater hydrophilicity of G, which makes it easier for funoran films to adsorb more water molecules [15,22]. The WVP values of the funoran films gradually increased with the addition of plasticizers, suggesting that the plasticizers reduced the intermolecular forces among funoran molecular chains. The free volume is expanded, and the movement of the funoran molecular chains is facilitated, allowing easier penetration of water molecules [24,41]. However, the prepared funoran films still have good water vapor barrier properties, which can be used for food packaging.

### 3.7. Light Transmittance Analysis

**Figure 9:**
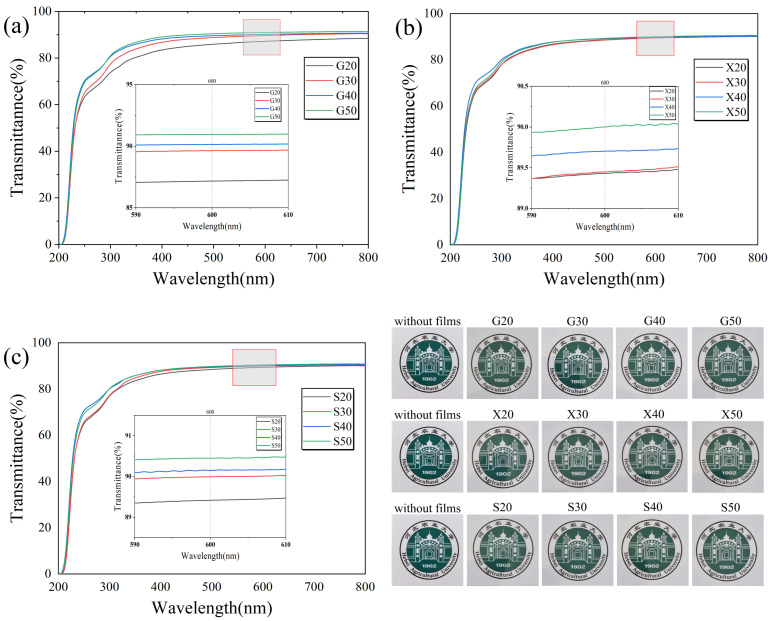
Effects of G (**a**), X (**b**) and S (**c**) addition on light transmission property of the funoran films. The Chinese in the figure refer to Hebei Agricultural University.

Light transmittance is one of the important indicators to determine whether a film can be used as food packaging [21,42]. The light transmittance curves of the funoran films with three different plasticizer additions are shown in Figure 9 and Table 1. It can be seen that the transmittance values of the funoran films increased with the addition of plasticizers. As the addition of G, X and S increased from 20% to 50%, the transmittance values at 600 nm increased from 87.16, 89.37, and 89.46 to 90.91, 89.88, and 90.58, respectively.

The light transmission of biopolymer films is strongly influenced by their spatial structure and the molecular weight of film-forming components [43,44]. Since the added plasticizers are hydrophilic, the absorption of water molecules increases with the addition of the plasticizer. Although the weakened interactions among funoran molecules increased the transmittance of the funoran films [39], the plasticized funoran films still have good transmittance values and can be used in food packaging.

## 4. Conclusions

In this study, a new funoran film was successfully developed by blending funoran with green plasticizers (G, X, and S). FTIR results demonstrated that the plasticizers altered the initial hydrogen bonds between funoran molecules, leading to the formation of new hydrogen bonds. SEM results revealed that the three plasticizers were compatible with funoran, and the prepared films were dense and homogeneous. TGA results showed that the thermal stability of the films decreased slightly with the addition of plasticizers, but all the funoran films remained stable at temperatures below 100 °C. Furthermore, the addition of plasticizers increased the EB, OP, WVP, and transmittance values of the funoran films but decreased the TS values. However, the maximum values of OP and WVP values were still at low levels. All the results demonstrated the good film-forming properties of funoran. The funoran film can reduce the water moisture and oxygen exchange between the external environment and food, inhibiting the growth and reproduction of microorganisms and extending the shelf life. This new edible film has a high potential for commercialization in the future.

## Figures and Tables

**Table 1:** Data summary table (TS, EB, OP, WVP, and Transmittance values).

Samples	TS (MPa)	EB (%)	OP × 10^−12^ (cm^3^∙cm)/(m^2^∙s∙0.1 MPa)	WVP × 10^−8^ g/(s∙m∙Pa)	Transmittance Value at 600 nm
G20	99.98 ± 2.12 ^cd^	18.35 ± 1.17 ^e^	7.73 ± 0.11 ^g^	3.80 ± 0.79 ^c^	87.16
G30	83.45 ± 0.96 ^b^	25.77 ± 0.88 ^f^	5.70 ± 0.18 ^f^	6.43 ± 0.99 ^d^	89.62
G40	60.32 ± 1.56 ^a^	36.53 ± 0.77 ^g^	5.18 ± 0.12 ^e^	6.35 ± 0.82 ^d^	90.17
G50	49.10 ± 1.14 ^a^	42.14 ± 0.51 ^h^	5.29 ± 0.05 ^e^	10.93 ± 0.55 ^f^	90.91
X20	124.48 ± 4.59 ^e^	5.57 ± 0.40 ^a^	5.76 ± 0.20 ^f^	1.59 ± 0.17 ^a^	89.37
X30	109.61 ± 10.39 ^d^	6.01 ± 0.40 ^ab^	5.50 ± 0.11 ^ef^	1.67 ± 0.18 ^a^	89.42
X40	106.97 ± 1.83 ^d^	7.20 ± 0.31 ^abc^	2.95 ± 0.04 ^bc^	2.34 ± 0.40 ^ab^	89.76
X50	88.77 ± 1.61 ^bc^	14.62 ± 2.04 ^d^	3.27 ± 0.01 ^cd^	2.99 ± 0.23 ^bc^	89.88
S20	112.04 ± 5.23 ^d^	5.25 ± 0.26 ^a^	3.33 ± 0.05 ^d^	1.69 ± 0.28 ^a^	89.46
S30	104.91 ± 4.47 ^d^	6.12 ± 0.57 ^ab^	2.81 ± 0.01 ^b^	1.71 ± 0.30 ^a^	89.97
S40	81.86 ± 1.09 ^b^	8.15 ± 0.43 ^bc^	2.74 ± 0.06 ^ab^	2.08 ± 0.04 ^a^	90.33
S50	78.90 ± 2.02 ^b^	9.40 ± 0.81 ^c^	2.44 ± 0.16 ^a^	1.75 ± 0.23 ^a^	90.59

Note: The values in each group with different superscript letter (a–h) indicates significant difference (*p* < 0.05).

## Data Availability

The data presented in this study are available within the article.
